# 1-Benzyl-3,5-bis­[(*E*)-3-thienyl­methyl­idene]piperidin-4-one

**DOI:** 10.1107/S1600536811003758

**Published:** 2011-02-05

**Authors:** P. Gayathri, A. Thiruvalluvar, K. Rajeswari, K. Pandiarajan, R. J. Butcher

**Affiliations:** a PG Research Department of Physics, Rajah Serfoji Government College (Autonomous), Thanjavur 613 005, Tamilnadu, India; b Department of Chemistry, Annamalai University, Annamalai Nagar 608 002, Tamilnadu, India; c Department of Chemistry, Howard University, 525 College Street NW, Washington, DC 20059, USA

## Abstract

In the title mol­ecule, C_22_H_19_NOS_2_, the piperidine ring adopts an envelope conformation with the benzyl substituent in an equatorial position. Each of the olefinic double bonds has an *E* configuration. The dihedral angle between the two thio­phene rings is 1.55 (18)°. The thio­phene rings form angles of 72.21 (14) and 73.43 (14)° with the phenyl ring. Both thio­phene rings are disordered over two orientations [occupancy ratios of 0.799 (1):0.201 (1)] at 180° from one another. In the crystal, weak inter­molecular C—H⋯O hydrogen bonds and C—H⋯π inter­actions help to stabilize the packing.

## Related literature

For a related structure and applications of piperidone derivatives, see: Rajeswari *et al.* (2009 ▶).
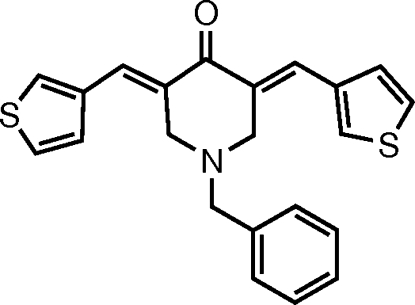


## Experimental

### 

#### Crystal data

C_22_H_19_NOS_2_
*M* *_r_* = 377.52
Triclinic, 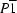

*a* = 6.1809 (3) Å
*b* = 12.7391 (9) Å
*c* = 12.9251 (7) Å
α = 112.657 (6)°
β = 95.845 (5)°
γ = 98.512 (5)°
*V* = 914.65 (11) Å^3^
*Z* = 2
Cu *K*α radiation
μ = 2.71 mm^−1^
*T* = 123 K
0.44 × 0.31 × 0.23 mm

#### Data collection

Oxford Diffraction Xcalibur Ruby Gemini diffractometer
Absorption correction: multi-scan (*CrysAlis PRO*; Oxford Diffraction, 2010 ▶) *T*_min_ = 0.587, *T*_max_ = 1.000
6091 measured reflections
3615 independent reflections
3114 reflections with *I* > 2σ(*I*)
*R*_int_ = 0.026

#### Refinement

*R*[*F* ^2^ > 2σ(*F* ^2^)] = 0.040
*wR*(*F* ^2^) = 0.110
*S* = 1.03
3615 reflections
276 parameters
72 restraints
H atoms treated by a mixture of independent and constrained refinement
Δρ_max_ = 0.37 e Å^−3^
Δρ_min_ = −0.27 e Å^−3^

### 

Data collection: *CrysAlis PRO* (Oxford Diffraction, 2010 ▶); cell refinement: *CrysAlis PRO*; data reduction: *CrysAlis RED* (Oxford Diffraction, 2010 ▶); program(s) used to solve structure: *SHELXS97* (Sheldrick, 2008 ▶); program(s) used to refine structure: *SHELXL97* (Sheldrick, 2008 ▶); molecular graphics: *ORTEP-3* (Farrugia, 1997 ▶) and *PLATON* (Spek, 2009 ▶); software used to prepare material for publication: *PLATON*.

## Supplementary Material

Crystal structure: contains datablocks global, I. DOI: 10.1107/S1600536811003758/jj2073sup1.cif

Structure factors: contains datablocks I. DOI: 10.1107/S1600536811003758/jj2073Isup2.hkl

Additional supplementary materials:  crystallographic information; 3D view; checkCIF report

## Figures and Tables

**Table 1:** Hydrogen-bond geometry (Å, °) *Cg*6 is the centroid of the C17–C22 ring.

*D*—H⋯*A*	*D*—H	H⋯*A*	*D*⋯*A*	*D*—H⋯*A*
C13*A*—H13*A*⋯O1^i^	0.95	2.39	3.177 (4)	140
C16—H16*A*⋯*Cg*6^ii^	0.99	2.68	3.6017 (19)	156

Symmetry codes: (i) 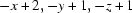; (ii) 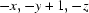.

**Fig. 1.**
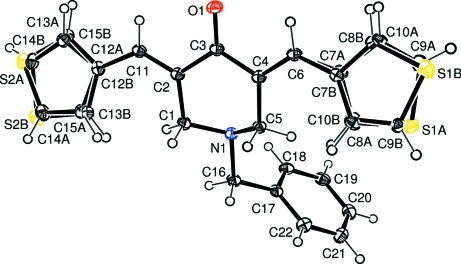
The molecular structure of the title compound, showing the atom-numbering scheme and displacement ellipsoids drawn at the 30% probability level. H atoms are shown as small spheres of arbitrary radius. Both thiophene rings are disordered over two orientations (0.799 (1)/0.201 (1)).

**Fig. 2.**
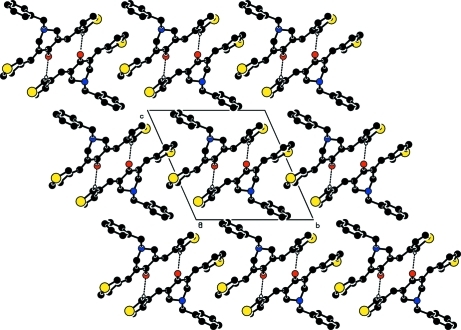
The packing of the title compound, viewed down the a axis. Dashed lines indicate weak hydrogen bonds. H atoms not involved in hydrogen bonding have been omitted.

## supplementary crystallographic information

### Comment

As part of our research (Rajeswari *et al.*, 2009), we have synthesized the title compound (I) and report its crystal structure here.

The molecular structure of the title compound (I) is shown in Fig. 1. The piperidine ring adopts an envelope conformation with the benzyl substituent in an equatorial position. The sum of the bond angles around N1 [328.38 (13)°] indicates a pyramidal geometry. The N1 atom deviates by 0.715 (2) Å from the least-squares plane passing through atoms C1—C5. Each of the olefinic double bonds has an E configuration. The dihedral angle between the two thiophene rings is 1.55 (18)°. The thiophene rings form angles of 72.21 (14) and 73.43 (14)° with the phenyl ring. Both thiophene rings are disordered over two orientations [occupancies of 0.799 (1)/0.201 (1)] at 180° from one another. In the crystal, weak intermolecular C13A—H13A···O1 hydrogen bonds and C16—H16A···π interactions involving the phenyl ring (C17—C22) help to stabilize the packing (Table 1, Fig. 2).

### Experimental

The mixture of 1-benzylpiperidin-4-one (1.9 ml, 0.01 mol) and thiophene-3-aldehyde (1.8 ml, 0.02 mol) in ethanol (95%, 10 ml), was added with 5 ml of 20% sodium hydroxide followed by the heating on a waterbath for 30 minutes. The solid separated was filtered and recrystallized by slow evaporation from 95% ethanol. The yield was (3.0 g, 80%).

### Refinement

H6 at C6 and H11 at C11 atoms were located in a difference Fourier map and refined freely: C6—H6 = 0.943 (19) Å, C11—H11 = 0.92 (2) Å. Remaining H atoms were positioned geomentrically and allowed to ride on their parent atoms, with C—H = 0.95 - 0.99 Å and *U*_iso_(H) = 1.2 times *U*_eq_(C). Both thiophene rings were found disordered with occupancies 0.799 (1)/0.201 (1). The disordered thiophene moieties were restricted to have C—S distances of 1.718 (2) to 1.718 (15) Å. A damping factor (damp 100 15 in the final refinement cycles) was applied to avoid large displacements of the hydrogen atoms of the less occupied thiophene rings.

### Crystal data

**Table:**

C_22_H_19_NOS_2_	*Z* = 2
*M**_r_* = 377.52	*F*(000) = 396
Triclinic, *P*1	*D*_x_ = 1.371 Mg m^−^^3^
Hall symbol: -P 1	Melting point: 429 K
*a* = 6.1809 (3) Å	Cu *K*α radiation, λ = 1.54184 Å
*b* = 12.7391 (9) Å	Cell parameters from 3996 reflections
*c* = 12.9251 (7) Å	θ = 6.4–74.0°
α = 112.657 (6)°	µ = 2.71 mm^−^^1^
β = 95.845 (5)°	*T* = 123 K
γ = 98.512 (5)°	Chunk, colourless
*V* = 914.65 (11) Å^3^	0.44 × 0.31 × 0.23 mm

### Data collection

**Table:**

Oxford Diffraction Xcalibur Ruby Gemini diffractometer	3615 independent reflections
Radiation source: Enhance (Cu) X-ray Source	3114 reflections with *I* > 2σ(*I*)
graphite	*R*_int_ = 0.026
Detector resolution: 10.5081 pixels mm^-1^	θ_max_ = 74.2°, θ_min_ = 6.4°
ω scans	*h* = −6→7
Absorption correction: multi-scan (*CrysAlis PRO*; Oxford Diffraction, 2010)	*k* = −14→15
*T*_min_ = 0.587, *T*_max_ = 1.000	*l* = −16→12
6091 measured reflections	

### Refinement

**Table:**

Refinement on *F*^2^	Primary atom site location: structure-invariant direct methods
Least-squares matrix: full	Secondary atom site location: difference Fourier map
*R*[*F*^2^ > 2σ(*F*^2^)] = 0.040	Hydrogen site location: inferred from neighbouring sites
*wR*(*F*^2^) = 0.110	H atoms treated by a mixture of independent and constrained refinement
*S* = 1.03	*w* = 1/[σ^2^(*F*_o_^2^) + (0.0635*P*)^2^ + 0.4312*P*] where *P* = (*F*_o_^2^ + 2*F*_c_^2^)/3
3615 reflections	(Δ/σ)_max_ = 0.001
276 parameters	Δρ_max_ = 0.37 e Å^−^^3^
72 restraints	Δρ_min_ = −0.27 e Å^−^^3^

### Special details

**Table:**

Geometry. Bond distances, angles *etc*. have been calculated using the rounded fractional coordinates. All su's are estimated from the variances of the (full) variance-covariance matrix. The cell e.s.d.'s are taken into account in the estimation of distances, angles and torsion angles
Refinement. Refinement of *F*^2^ against ALL reflections. The weighted *R*-factor *wR* and goodness of fit *S* are based on *F*^2^, conventional *R*-factors *R* are based on *F*, with *F* set to zero for negative *F*^2^. The threshold expression of *F*^2^ > 2σ(*F*^2^) is used only for calculating *R*-factors(gt) *etc*. and is not relevant to the choice of reflections for refinement. *R*-factors based on *F*^2^ are statistically about twice as large as those based on *F*, and *R*- factors based on ALL data will be even larger.

### Fractional atomic coordinates and isotropic or equivalent isotropic displacement parameters (Å^2^)

**Table:**

	*x*	*y*	*z*	*U*_iso_*/*U*_eq_	Occ. (<1)
S1A	0.18315 (9)	1.03620 (4)	0.61944 (5)	0.0240 (2)	0.799 (1)
S2A	0.73267 (10)	0.08626 (5)	0.16709 (6)	0.0279 (2)	0.799 (1)
O1	0.8340 (2)	0.63874 (10)	0.50798 (10)	0.0306 (3)	
N1	0.3176 (2)	0.57437 (11)	0.26094 (11)	0.0217 (4)	
C1	0.3584 (3)	0.46955 (14)	0.27383 (14)	0.0246 (4)	
C2	0.5717 (3)	0.49305 (13)	0.35403 (13)	0.0214 (4)	
C3	0.6540 (3)	0.61272 (14)	0.44395 (13)	0.0220 (4)	
C4	0.5080 (3)	0.69853 (13)	0.45572 (13)	0.0201 (4)	
C5	0.2948 (3)	0.66158 (13)	0.37148 (13)	0.0212 (4)	
C6	0.5750 (3)	0.80356 (12)	0.54314 (13)	0.0210 (4)	
C7A	0.4685 (3)	0.90348 (14)	0.5796 (3)	0.0183 (5)	0.799 (1)
C8A	0.2461 (4)	0.90208 (14)	0.5516 (3)	0.0207 (6)	0.799 (1)
C9A	0.4535 (3)	1.0966 (3)	0.6871 (4)	0.0329 (4)	0.799 (1)
C10A	0.5831 (4)	1.0177 (2)	0.6602 (3)	0.0234 (6)	0.799 (1)
C11	0.6925 (3)	0.41222 (11)	0.34934 (12)	0.0223 (4)	
C12A	0.6495 (7)	0.28977 (15)	0.2719 (3)	0.0228 (5)	0.799 (1)
C13A	0.8188 (6)	0.22914 (15)	0.2613 (3)	0.0238 (7)	0.799 (1)
C14A	0.4666 (3)	0.1060 (3)	0.1415 (4)	0.0310 (5)	0.799 (1)
C15A	0.4455 (6)	0.2152 (2)	0.2014 (4)	0.0263 (6)	0.799 (1)
C16	0.1124 (3)	0.54282 (14)	0.17762 (14)	0.0250 (4)	
C17	0.0842 (3)	0.63387 (14)	0.13266 (13)	0.0231 (4)	
C18	0.2395 (3)	0.66337 (15)	0.07197 (14)	0.0276 (5)	
C19	0.2113 (3)	0.74264 (16)	0.02500 (15)	0.0310 (5)	
C20	0.0256 (3)	0.79278 (16)	0.03671 (15)	0.0320 (5)	
C21	−0.1304 (3)	0.76399 (16)	0.09586 (15)	0.0325 (5)	
C22	−0.1007 (3)	0.68515 (15)	0.14398 (14)	0.0278 (5)	
C8B	0.5804 (14)	1.0004 (7)	0.6512 (15)	0.0207 (6)	0.201 (1)
C9B	0.1915 (10)	1.0076 (7)	0.6059 (10)	0.0240 (2)	0.201 (1)
S1B	0.4253 (4)	1.1062 (3)	0.6913 (4)	0.0329 (4)	0.201 (1)
S2B	0.4472 (4)	0.0861 (3)	0.1254 (4)	0.0310 (5)	0.201 (1)
C13B	0.438 (2)	0.2290 (5)	0.2021 (17)	0.0238 (7)	0.201 (1)
C14B	0.7253 (7)	0.1115 (10)	0.1784 (12)	0.0279 (2)	0.201 (1)
C7B	0.4467 (8)	0.8937 (4)	0.5864 (15)	0.0183 (5)	0.201 (1)
C12B	0.640 (3)	0.2900 (3)	0.2707 (10)	0.0228 (5)	0.201 (1)
C10B	0.221 (2)	0.9007 (8)	0.5623 (14)	0.0234 (6)	0.201 (1)
C15B	0.800 (3)	0.2159 (10)	0.2558 (16)	0.0263 (6)	0.201 (1)
H1B	0.36535	0.40961	0.19840	0.0296*	
H5A	0.25596	0.73017	0.36152	0.0255*	
H5B	0.17305	0.62863	0.40123	0.0255*	
H1A	0.23312	0.43872	0.30305	0.0296*	
H16A	0.11277	0.46894	0.11292	0.0300*	
H16B	−0.01661	0.52956	0.21384	0.0300*	
H18	0.36607	0.62866	0.06278	0.0331*	
H19	0.31915	0.76265	−0.01525	0.0372*	
H20	0.00581	0.84676	0.00415	0.0383*	
H21	−0.25789	0.79798	0.10372	0.0390*	
H22	−0.20800	0.66618	0.18501	0.0333*	
H6	0.713 (3)	0.8169 (16)	0.5895 (17)	0.026 (5)*	
H8A	0.14054	0.83497	0.50085	0.0249*	0.799 (1)
H9A	0.50486	1.17598	0.73726	0.0395*	0.799 (1)
H10A	0.73660	1.03548	0.69141	0.0281*	0.799 (1)
H11	0.823 (3)	0.4373 (16)	0.4015 (17)	0.026 (5)*	
H13A	0.96646	0.26254	0.30162	0.0286*	0.799 (1)
H14A	0.34781	0.04620	0.09052	0.0372*	0.799 (1)
H15A	0.30930	0.24073	0.19759	0.0316*	0.799 (1)
H8B	0.73734	1.01388	0.67126	0.0249*	0.201 (1)
H9B	0.05409	1.02885	0.59182	0.0288*	0.201 (1)
H10B	0.10440	0.83526	0.51955	0.0281*	0.201 (1)
H13B	0.31035	0.26169	0.19782	0.0286*	0.201 (1)
H14B	0.81485	0.05504	0.15365	0.0335*	0.201 (1)
H15B	0.94614	0.24115	0.29862	0.0316*	0.201 (1)

### Atomic displacement parameters (Å^2^)

**Table:**

	*U*^11^	*U*^22^	*U*^33^	*U*^12^	*U*^13^	*U*^23^
S1A	0.0245 (2)	0.0238 (3)	0.0207 (3)	0.0093 (2)	0.0021 (2)	0.0046 (2)
S2A	0.0394 (3)	0.0223 (3)	0.0221 (3)	0.0103 (2)	0.0069 (2)	0.0073 (2)
O1	0.0245 (6)	0.0280 (6)	0.0298 (6)	0.0071 (5)	−0.0056 (5)	0.0036 (5)
N1	0.0241 (7)	0.0213 (6)	0.0165 (6)	0.0037 (5)	−0.0021 (5)	0.0060 (5)
C1	0.0267 (8)	0.0213 (7)	0.0214 (8)	0.0028 (6)	−0.0019 (6)	0.0061 (6)
C2	0.0223 (7)	0.0236 (8)	0.0174 (7)	0.0032 (6)	0.0026 (6)	0.0081 (6)
C3	0.0215 (8)	0.0249 (8)	0.0182 (7)	0.0035 (6)	0.0017 (6)	0.0083 (6)
C4	0.0206 (7)	0.0233 (7)	0.0170 (7)	0.0039 (6)	0.0028 (6)	0.0092 (6)
C5	0.0208 (7)	0.0231 (7)	0.0182 (7)	0.0044 (6)	0.0010 (6)	0.0074 (6)
C6	0.0193 (7)	0.0258 (8)	0.0178 (7)	0.0036 (6)	0.0016 (6)	0.0094 (6)
C7A	0.0202 (8)	0.0218 (8)	0.0143 (8)	0.0006 (6)	0.0049 (7)	0.0098 (7)
C8A	0.0215 (11)	0.0253 (10)	0.0147 (11)	0.0062 (8)	0.0045 (8)	0.0066 (8)
C9A	0.0376 (8)	0.0298 (7)	0.0286 (6)	0.0031 (6)	0.0043 (7)	0.0106 (5)
C10A	0.0225 (10)	0.0219 (11)	0.0185 (12)	−0.0006 (9)	0.0023 (8)	0.0025 (10)
C11	0.0221 (8)	0.0246 (8)	0.0185 (7)	0.0025 (6)	0.0017 (6)	0.0081 (6)
C12A	0.0290 (9)	0.0229 (8)	0.0173 (7)	0.0048 (6)	0.0044 (6)	0.0090 (6)
C13A	0.0308 (13)	0.0199 (11)	0.0208 (10)	0.0054 (10)	0.0040 (9)	0.0083 (9)
C14A	0.0412 (7)	0.0237 (9)	0.0216 (10)	0.0030 (6)	0.0020 (5)	0.0046 (7)
C15A	0.0285 (11)	0.0264 (11)	0.0248 (10)	0.0041 (10)	0.0016 (9)	0.0125 (11)
C16	0.0254 (8)	0.0253 (8)	0.0193 (7)	0.0011 (6)	−0.0047 (6)	0.0072 (6)
C17	0.0258 (8)	0.0224 (7)	0.0147 (7)	0.0009 (6)	−0.0047 (6)	0.0043 (6)
C18	0.0234 (8)	0.0327 (9)	0.0225 (8)	0.0031 (7)	−0.0017 (6)	0.0091 (7)
C19	0.0292 (9)	0.0368 (9)	0.0244 (8)	−0.0019 (7)	−0.0007 (7)	0.0141 (7)
C20	0.0406 (10)	0.0284 (8)	0.0251 (8)	0.0025 (7)	−0.0032 (7)	0.0127 (7)
C21	0.0360 (10)	0.0328 (9)	0.0286 (9)	0.0132 (8)	0.0037 (7)	0.0106 (7)
C22	0.0295 (9)	0.0327 (9)	0.0219 (8)	0.0076 (7)	0.0054 (7)	0.0113 (7)
C8B	0.0215 (11)	0.0253 (10)	0.0147 (11)	0.0062 (8)	0.0045 (8)	0.0066 (8)
C9B	0.0245 (2)	0.0238 (3)	0.0207 (3)	0.0093 (2)	0.0021 (2)	0.0046 (2)
S1B	0.0376 (8)	0.0298 (7)	0.0286 (6)	0.0031 (6)	0.0043 (7)	0.0106 (5)
S2B	0.0412 (7)	0.0237 (9)	0.0216 (10)	0.0030 (6)	0.0020 (5)	0.0046 (7)
C13B	0.0308 (13)	0.0199 (11)	0.0208 (10)	0.0054 (10)	0.0040 (9)	0.0083 (9)
C14B	0.0394 (3)	0.0223 (3)	0.0221 (3)	0.0103 (2)	0.0069 (2)	0.0073 (2)
C7B	0.0202 (8)	0.0218 (8)	0.0143 (8)	0.0006 (6)	0.0049 (7)	0.0098 (7)
C12B	0.0290 (9)	0.0229 (8)	0.0173 (7)	0.0048 (6)	0.0044 (6)	0.0090 (6)
C10B	0.0225 (10)	0.0219 (11)	0.0185 (12)	−0.0006 (9)	0.0023 (8)	0.0025 (10)
C15B	0.0285 (11)	0.0264 (11)	0.0248 (10)	0.0041 (10)	0.0016 (9)	0.0125 (11)

### Geometric parameters (Å, °)

**Table:**

S1A—C8A	1.718 (3)	C14B—C15B	1.30 (2)
S1A—C9A	1.718 (3)	C16—C17	1.507 (3)
S1B—C9B	1.719 (10)	C17—C18	1.397 (3)
S1B—C8B	1.718 (12)	C17—C22	1.391 (3)
S2A—C13A	1.718 (3)	C18—C19	1.386 (3)
S2A—C14A	1.718 (2)	C19—C20	1.388 (3)
S2B—C13B	1.718 (15)	C20—C21	1.382 (3)
S2B—C14B	1.719 (8)	C21—C22	1.394 (3)
O1—C3	1.235 (2)	C1—H1A	0.9900
N1—C5	1.470 (2)	C1—H1B	0.9900
N1—C16	1.472 (2)	C5—H5A	0.9900
N1—C1	1.462 (2)	C5—H5B	0.9900
C1—C2	1.506 (3)	C6—H6	0.943 (19)
C2—C3	1.490 (2)	C8A—H8A	0.9500
C2—C11	1.345 (2)	C8B—H8B	0.9500
C3—C4	1.491 (3)	C9A—H9A	0.9500
C4—C6	1.347 (2)	C9B—H9B	0.9500
C4—C5	1.508 (2)	C10A—H10A	0.9500
C6—C7A	1.458 (3)	C10B—H10B	0.9500
C6—C7B	1.458 (9)	C11—H11	0.92 (2)
C7A—C8A	1.381 (3)	C13A—H13A	0.9500
C7A—C10A	1.443 (4)	C13B—H13B	0.9500
C7B—C8B	1.372 (18)	C14A—H14A	0.9500
C7B—C10B	1.422 (14)	C14B—H14B	0.9500
C9A—C10A	1.341 (4)	C15A—H15A	0.9500
C9B—C10B	1.307 (17)	C15B—H15B	0.9500
C11—C12A	1.458 (3)	C16—H16A	0.9900
C11—C12B	1.458 (9)	C16—H16B	0.9900
C12A—C15A	1.438 (6)	C18—H18	0.9500
C12A—C13A	1.377 (5)	C19—H19	0.9500
C12B—C13B	1.38 (2)	C20—H20	0.9500
C12B—C15B	1.44 (2)	C21—H21	0.9500
C14A—C15A	1.340 (6)	C22—H22	0.9500
			
S1A···C8A^i^	3.648 (3)	C9A···H5A^iii^	2.9400
S1A···S1A^i^	3.3576 (9)	C9A···H21^i^	2.9900
S1A···S2A^ii^	3.6853 (9)	C9B···H9B^i^	2.6700
S1A···C21^i^	3.6606 (19)	C9B···H8B^v^	3.0200
S1B···C21^i^	3.469 (4)	C10B···H9B^i^	2.9700
S1B···C6^iii^	3.631 (5)	C10B···H5A	2.7200
S1B···C18^iii^	3.502 (5)	C12A···H1B	2.8100
S1B···C5^iii^	3.609 (5)	C12B···H1B	2.7600
S1B···C4^iii^	3.670 (5)	C13B···H19^vi^	3.0000
S2A···C9A^ii^	3.651 (4)	C13B···H1B	2.4300
S2A···S1A^ii^	3.6853 (9)	C13B···H1A	3.0200
S2A···C10A^ii^	3.598 (3)	C14A···H19^vi^	3.0200
S2A···C8A^ii^	3.569 (3)	C15A···H1B	2.6100
S2A···C7A^ii^	3.582 (3)	C15A···H19^vi^	3.0200
S2B···C14B^iv^	3.692 (15)	C17···H5A	2.7500
S2B···C8B^ii^	3.475 (16)	C17···H16A^ix^	2.9700
S2B···S2B^iv^	3.335 (6)	C17···H9A^iii^	3.0500
S2B···C7B^ii^	3.618 (17)	C18···H16A^ix^	2.8400
S1A···H10A^v^	3.0000	C18···H9A^iii^	2.6700
S1B···H21^i^	2.8300	C19···H16A^ix^	2.9000
S1B···H5A^iii^	2.9700	C19···H9A^iii^	3.0800
S2A···H20^vi^	3.1600	C20···H16A^ix^	3.0700
S2A···H14A^iv^	3.0400	H1A···H13A^v^	2.5700
S2B···H19^vi^	3.0900	H1A···H16B	2.5000
O1···C15B^vii^	3.296 (19)	H1A···C13B	3.0200
O1···C13A^vii^	3.177 (4)	H1A···H5B	2.3700
O1···H10B^viii^	2.7400	H1A···H15A	2.5000
O1···H11^vii^	2.79 (2)	H1A···H13B	2.2900
O1···H13A^vii^	2.3900	H1B···C15A	2.6100
O1···H6	2.38 (2)	H1B···H15A	2.1200
O1···H11	2.39 (2)	H1B···C13B	2.4300
O1···H5B^viii^	2.6200	H1B···H13B	1.8600
O1···H15B^vii^	2.4900	H1B···C12A	2.8100
N1···H18	2.9300	H1B···C12B	2.7600
C1···C15A	3.154 (4)	H1B···H16A	2.2100
C1···C13B	2.974 (12)	H5A···C10B	2.7200
C4···C11^ii^	3.568 (2)	H5A···C7B	2.8400
C4···S1B^iii^	3.670 (5)	H5A···C7A	2.8500
C5···C10B	3.228 (15)	H5A···C8A	2.6000
C5···S1B^iii^	3.609 (5)	H5A···C17	2.7500
C5···C8A	3.133 (3)	H5A···H8A	2.0600
C6···C15A^ii^	3.415 (5)	H5A···C9A^iii^	2.9400
C6···C12B^ii^	3.372 (12)	H5A···S1B^iii^	2.9700
C6···S1B^iii^	3.631 (5)	H5A···H10B	2.3400
C6···C13B^ii^	3.481 (18)	H5A···H9A^iii^	2.5100
C6···C12A^ii^	3.381 (4)	H5B···H1A	2.3700
C7A···C13A^ii^	3.573 (4)	H5B···H8A	2.4900
C7A···C9A^iii^	3.529 (6)	H5B···O1^v^	2.6200
C7A···S2A^ii^	3.582 (3)	H5B···H16B	2.3400
C7B···S2B^ii^	3.618 (17)	H6···O1	2.38 (2)
C7B···C15B^ii^	3.26 (2)	H6···H10A	2.5500
C7B···C14B^ii^	3.34 (2)	H6···H8B	2.2900
C7B···C12B^ii^	3.512 (14)	H8A···H5B	2.4900
C8A···S2A^ii^	3.569 (3)	H8A···H5A	2.0600
C8A···C5	3.133 (3)	H8A···C4	3.0300
C8A···C13A^ii^	3.452 (4)	H8A···C5	2.5900
C8A···C10A^iii^	3.475 (4)	H8B···C9B^viii^	3.0200
C8A···S1A^i^	3.648 (3)	H8B···H6	2.2900
C8B···S2B^ii^	3.475 (16)	H8B···H9B^viii^	2.3200
C8B···C14B^ii^	3.595 (18)	H9A···C17^iii^	3.0500
C9A···C18^iii^	3.520 (5)	H9A···H5A^iii^	2.5100
C9A···S2A^ii^	3.651 (4)	H9A···C19^iii^	3.0800
C9A···C7A^iii^	3.529 (6)	H9A···C18^iii^	2.6700
C9B···C9B^i^	3.357 (14)	H9B···H9B^i^	2.1900
C10A···C14A^ii^	3.504 (6)	H9B···H8B^v^	2.3200
C10A···C8A^iii^	3.475 (4)	H9B···C10B^i^	2.9700
C10A···S2A^ii^	3.598 (3)	H9B···C9B^i^	2.6700
C10B···C15B^ii^	3.23 (2)	H10A···H6	2.5500
C10B···C14B^ii^	3.40 (2)	H10A···S1A^viii^	3.0000
C10B···C5	3.228 (15)	H10B···H5A	2.3400
C11···C4^ii^	3.568 (2)	H10B···C5	2.8100
C12A···C6^ii^	3.381 (4)	H10B···O1^v^	2.7400
C12B···C6^ii^	3.372 (12)	H11···O1^vii^	2.79 (2)
C12B···C7B^ii^	3.512 (14)	H11···O1	2.39 (2)
C13A···C8A^ii^	3.452 (4)	H11···H13A	2.4500
C13A···O1^vii^	3.177 (4)	H13A···O1^vii^	2.3900
C13A···C7A^ii^	3.573 (4)	H13A···H1A^viii^	2.5700
C13B···C6^ii^	3.481 (18)	H13A···H11	2.4500
C13B···C1	2.974 (12)	H13B···H1B	1.8600
C14A···C10A^ii^	3.504 (6)	H13B···C2	2.9500
C14B···C7B^ii^	3.34 (2)	H13B···H1A	2.2900
C14B···C10B^ii^	3.40 (2)	H13B···C1	2.4000
C14B···C8B^ii^	3.595 (18)	H14A···S2A^iv^	3.0400
C14B···S2B^iv^	3.692 (15)	H15A···C1	2.6500
C15A···C6^ii^	3.415 (5)	H15A···H1B	2.1200
C15A···C1	3.154 (4)	H15A···H1A	2.5000
C15B···C10B^ii^	3.23 (2)	H15B···O1^vii^	2.4900
C15B···O1^vii^	3.296 (19)	H16A···C17^ix^	2.9700
C15B···C7B^ii^	3.26 (2)	H16A···H1B	2.2100
C16···C18^ix^	3.520 (2)	H16A···C20^ix^	3.0700
C18···C16^ix^	3.520 (2)	H16A···C18^ix^	2.8400
C18···S1B^iii^	3.502 (5)	H16A···C19^ix^	2.9000
C18···C9A^iii^	3.520 (5)	H16B···H22	2.3600
C21···S1B^i^	3.469 (4)	H16B···H1A	2.5000
C21···S1A^i^	3.6606 (19)	H16B···H5B	2.3400
C1···H13B	2.4000	H18···N1	2.9300
C1···H15A	2.6500	H19···S2B^vi^	3.0900
C2···H13B	2.9500	H19···C14A^vi^	3.0200
C4···H8A	3.0300	H19···C15A^vi^	3.0200
C5···H10B	2.8100	H19···C13B^vi^	3.0000
C5···H8A	2.5900	H20···S2A^vi^	3.1600
C7A···H5A	2.8500	H21···S1B^i^	2.8300
C7B···H5A	2.8400	H21···C9A^i^	2.9900
C8A···H5A	2.6000	H22···H16B	2.3600
			
C8A—S1A—C9A	92.00 (17)	N1—C1—H1A	109.00
C8B—S1B—C9B	90.4 (6)	N1—C1—H1B	109.00
C13A—S2A—C14A	91.2 (2)	C2—C1—H1A	109.00
C13B—S2B—C14B	91.2 (7)	C2—C1—H1B	109.00
C1—N1—C5	109.61 (13)	H1A—C1—H1B	108.00
C5—N1—C16	110.43 (13)	N1—C5—H5A	109.00
C1—N1—C16	108.34 (14)	N1—C5—H5B	109.00
N1—C1—C2	111.86 (15)	C4—C5—H5A	109.00
C1—C2—C3	118.18 (16)	C4—C5—H5B	109.00
C1—C2—C11	123.85 (15)	H5A—C5—H5B	108.00
C3—C2—C11	117.97 (15)	C4—C6—H6	116.8 (13)
O1—C3—C2	121.03 (17)	C7A—C6—H6	112.8 (13)
C2—C3—C4	117.50 (15)	C7B—C6—H6	114.6 (14)
O1—C3—C4	121.44 (16)	S1A—C8A—H8A	124.00
C5—C4—C6	124.57 (17)	C7A—C8A—H8A	124.00
C3—C4—C6	117.12 (16)	C7B—C8B—H8B	125.00
C3—C4—C5	118.29 (14)	S1B—C8B—H8B	125.00
N1—C5—C4	110.75 (14)	C10A—C9A—H9A	124.00
C4—C6—C7A	130.47 (19)	S1A—C9A—H9A	124.00
C4—C6—C7B	127.9 (5)	C10B—C9B—H9B	123.00
C6—C7A—C8A	126.3 (2)	S1B—C9B—H9B	123.00
C8A—C7A—C10A	110.5 (2)	C9A—C10A—H10A	123.00
C6—C7A—C10A	123.0 (2)	C7A—C10A—H10A	123.00
C6—C7B—C10B	134.6 (12)	C9B—C10B—H10B	124.00
C6—C7B—C8B	112.0 (6)	C7B—C10B—H10B	124.00
C8B—C7B—C10B	112.4 (9)	C12A—C11—H11	114.6 (13)
S1A—C8A—C7A	111.9 (2)	C2—C11—H11	116.2 (13)
S1B—C8B—C7B	110.7 (7)	C12B—C11—H11	116.8 (15)
S1A—C9A—C10A	111.6 (3)	C12A—C13A—H13A	124.00
S1B—C9B—C10B	113.6 (8)	S2A—C13A—H13A	124.00
C7A—C10A—C9A	114.0 (3)	S2B—C13B—H13B	124.00
C7B—C10B—C9B	112.2 (11)	C12B—C13B—H13B	125.00
C2—C11—C12A	129.3 (2)	S2A—C14A—H14A	124.00
C2—C11—C12B	127.0 (7)	C15A—C14A—H14A	124.00
C11—C12A—C13A	119.8 (3)	S2B—C14B—H14B	124.00
C11—C12A—C15A	129.5 (3)	C15B—C14B—H14B	124.00
C13A—C12A—C15A	110.7 (3)	C12A—C15A—H15A	123.00
C13B—C12B—C15B	110.8 (11)	C14A—C15A—H15A	123.00
C11—C12B—C13B	126.5 (13)	C12B—C15B—H15B	123.00
C11—C12B—C15B	122.8 (14)	C14B—C15B—H15B	123.00
S2A—C13A—C12A	112.4 (3)	H16A—C16—H16B	108.00
S2B—C13B—C12B	111.1 (9)	N1—C16—H16B	109.00
S2A—C14A—C15A	112.4 (3)	C17—C16—H16A	109.00
S2B—C14B—C15B	112.9 (10)	N1—C16—H16A	109.00
C12A—C15A—C14A	113.3 (3)	C17—C16—H16B	109.00
C12B—C15B—C14B	113.7 (14)	C17—C18—H18	120.00
N1—C16—C17	113.51 (15)	C19—C18—H18	120.00
C18—C17—C22	118.42 (17)	C18—C19—H19	120.00
C16—C17—C18	119.81 (17)	C20—C19—H19	120.00
C16—C17—C22	121.66 (16)	C19—C20—H20	120.00
C17—C18—C19	120.85 (17)	C21—C20—H20	120.00
C18—C19—C20	120.05 (18)	C20—C21—H21	120.00
C19—C20—C21	119.84 (19)	C22—C21—H21	120.00
C20—C21—C22	120.03 (18)	C21—C22—H22	120.00
C17—C22—C21	120.80 (17)	C17—C22—H22	120.00
			
C9A—S1A—C8A—C7A	−0.2 (3)	C6—C4—C5—N1	151.56 (17)
C8A—S1A—C9A—C10A	−0.9 (3)	C4—C6—C7A—C8A	19.5 (4)
C14A—S2A—C13A—C12A	−0.4 (3)	C4—C6—C7A—C10A	−166.4 (3)
C13A—S2A—C14A—C15A	0.0 (4)	C10A—C7A—C8A—S1A	1.2 (3)
C5—N1—C1—C2	−62.00 (18)	C6—C7A—C8A—S1A	175.9 (2)
C16—N1—C1—C2	177.45 (13)	C6—C7A—C10A—C9A	−176.8 (3)
C16—N1—C5—C4	−177.33 (14)	C8A—C7A—C10A—C9A	−1.9 (4)
C1—N1—C16—C17	−164.29 (14)	S1A—C9A—C10A—C7A	1.7 (4)
C5—N1—C16—C17	75.66 (18)	C2—C11—C12A—C15A	−18.5 (5)
C1—N1—C5—C4	63.39 (18)	C2—C11—C12A—C13A	163.1 (2)
N1—C1—C2—C11	−153.36 (16)	C15A—C12A—C13A—S2A	0.7 (4)
N1—C1—C2—C3	26.7 (2)	C11—C12A—C13A—S2A	179.4 (2)
C11—C2—C3—C4	−174.12 (16)	C11—C12A—C15A—C14A	−179.2 (3)
C3—C2—C11—C12A	179.2 (2)	C13A—C12A—C15A—C14A	−0.7 (5)
C11—C2—C3—O1	4.2 (3)	S2A—C14A—C15A—C12A	0.4 (5)
C1—C2—C11—C12A	−0.8 (3)	N1—C16—C17—C18	62.1 (2)
C1—C2—C3—C4	5.8 (2)	N1—C16—C17—C22	−121.80 (17)
C1—C2—C3—O1	−175.89 (16)	C16—C17—C18—C19	176.81 (16)
O1—C3—C4—C5	177.70 (16)	C22—C17—C18—C19	0.6 (3)
O1—C3—C4—C6	−3.6 (3)	C16—C17—C22—C21	−176.10 (16)
C2—C3—C4—C5	−4.0 (2)	C18—C17—C22—C21	0.1 (3)
C2—C3—C4—C6	174.63 (16)	C17—C18—C19—C20	−0.8 (3)
C3—C4—C5—N1	−29.9 (2)	C18—C19—C20—C21	0.4 (3)
C3—C4—C6—C7A	−177.8 (2)	C19—C20—C21—C22	0.3 (3)
C5—C4—C6—C7A	0.7 (3)	C20—C21—C22—C17	−0.5 (3)

Symmetry codes: (i) −*x*, −*y*+2, −*z*+1; (ii) −*x*+1, −*y*+1, −*z*+1; (iii) −*x*+1, −*y*+2, −*z*+1; (iv) −*x*+1, −*y*, −*z*; (v) *x*−1, *y*, *z*; (vi) −*x*+1, −*y*+1, −*z*; (vii) −*x*+2, −*y*+1, −*z*+1; (viii) *x*+1, *y*, *z*; (ix) −*x*, −*y*+1, −*z*.

### Hydrogen-bond geometry (Å, °)

**Table:**

Cg6 is the centroid of the C17–C22 ring.

**Table:**

*D*—H···*A*	*D*—H	H···*A*	*D*···*A*	*D*—H···*A*
C13A—H13A···O1^vii^	0.95	2.39	3.177 (4)	140
C16—H16A···Cg6^ix^	0.99	2.68	3.6017 (19)	156

Symmetry codes: (vii) −*x*+2, −*y*+1, −*z*+1; (ix) −*x*, −*y*+1, −*z*.
